# Nafamostat mesylate, a serine protease inhibitor, demonstrates novel antimicrobial properties and effectiveness in Chlamydia-induced arthritis

**DOI:** 10.1186/ar3886

**Published:** 2012-06-20

**Authors:** Robert D Inman, Basil Chiu

**Affiliations:** 1 Division of Genetics and Development, Toronto Western Research Institute, 399 Bathurst Street, Toronto, M5T 2S8, Canada

## Abstract

**Introduction:**

Effective treatment of reactive arthritis would ideally achieve both control of inflammation and eradication of persisting arthritogenic pathogens. We use a model of experimental *Chlamydia trachomatis*-induced arthritis (CtIA) to evaluate the effectiveness of nafamostat mesilate (NM), a serine protease inhibitor with complement-modifying effects and anticoagulant properties. To date clinical use of NM has largely been in Asia and has been primarily confined to inflammatory states such as pancreatitis.

**Methods:**

*In vitro *studies examined inhibition of *Chlamydia *proliferation using fibroblast cell lines as targets and phase contrast microscopy. *In vivo *studies used an established protocol, experimental CtIA, induced in Lewis rats by injection of synoviocyte-packaged *C. trachomatis*. NM was dissolved in water and administered by daily intraperitoneal injection at a dose of 10 mg/kg beginning the day prior to the administration of *Chlamydia*. Readouts *in vivo *included (i) joint swelling, (ii) histopathology scoring of severity of arthritis, (iii) host clearance of the pathogen (by ELISA, the IDEIA *PCE *Chlamydia).

**Results:**

NM exerted a dose-dependent inhibition of chlamydial proliferation *in vitro*. Without NM, the mean number of inclusion bodies (IB) per well was 17,886 (± 1415). At 5 μg/mL NM, there were 8,490 (± 756) IB, at 25 μg/mL NM there were 35 IB and at 50 μg/mL NM no IB was observed. Chlamydial antigens in each well along the concentration gradient were assayed by ELISA, demonstrating that at 25 μg/mL NM inhibition of *Chlamydia *was almost complete. In the experimental arthritis model, joint swelling was significantly reduced with NM treatment: average joint width for the NM-treated animals was 8.55 mm (s.d. ± 0.6578, *n *= 10) *versus *11.18 mm (s.d. ± 0.5672, *n *= 10) in controls (*P *< 0.001). Histopathology scoring indicated that NM resulted in a marked attenuation of the inflammatory infiltration and joint damage: mean pathology score in NM-treated animals was 10.9 (± 2.45, *n *= 11) *versus *15.9 (± 1.45, *n *= 10) in controls (*P *< 0.0001). With respect to persistence of *Chlamydia *within the synovial tissues, NM treatment was accompanied by a reduction in the microbial load in the joint: mean optical density (O.D.) for ELISA with NM treatment was 0.05 (± 0.02, *n *= 4) *versus *0.18 (± 0.05, *n *= 4) in controls (*P *< 0.001).

**Conclusions:**

NM is a protease inhibitor not previously recognized to possess antimicrobial properties. The present study demonstrates for the first time that NM exerts significant impact on *C. trachomatis*-induced arthritis and suggests that such approaches may prove clinically useful in chronic reactive arthritis.

## Introduction

The pathogenesis of reactive arthritis triggered by a *Chlamydia trachomatis *infection has remained difficult to define in the clinical setting [1]. We have established a model of *C. trachomatis*-induced arthritis (CtIA) in rats in which live *Chlamydia *was grown up in carrier synovial fibroblasts and then injected directly into the knee joints of rats [2]. We have defined the importance of the cytokine profile in the development of CtIA [3] and have also addressed the role of complement in the resulting joint inflammatory process. To this end we attempted to modify joint inflammation by administrating cobra venom factor to decomplement the animals but no significant effect was seen. In our search for other complement-modifying agents, we have examined a synthetic compound, nafamostat mesylate (NM) for its effect on CtIA. NM, formerly known by the name FUT-175, is a serine protease inhibitor that has been used clinically in Asia, mainly Japan, as an anti-inflammatory agent [4,5]. It is generally well tolerated although rare occurrences of allergic reactions, hyperkalemia and hemolysis have been reported. It has been used for its complement-modifying effect and anticoagulant properties in the treatment of pancreatitis and disseminated intravascular coagulation. To date there are no data addressing the antimicrobial effects of NM, nor its effect on reactive arthritis.

## Materials and methods

### Rats

Eight-week old male Lewis rats were purchased from Harlan Laboratories (Indianapolis, IN, USA). They were maintained in microisolators under specific pathogen-free conditions in the animal care facility of the Toronto Western Hospital, University Health Network. All studies were conducted with the approval of the Animal Care Committee of the University Health Network.

### Induction of arthritis

Arthritis was induced in the rats by the intra-articular injection of live *Chlamydia *packaged in Lewis rat synovial fibroblasts as previously described [2,3]. Briefly, *C. trachomatis *serotype L2 was inoculated into monolayers of the fibroblast lines in culture. After overnight incubation, the cells containing chlamydia were harvested and injected into the knee joint of each rat at 5 × 10^5 ^colony forming units (CFU)/joint. Rats were assessed on a daily basis and then sacrificed four days after injection. At necropsy, their knee joints were either processed for histopathology or for quantitation of intra-articular *Chlamydia*.

### Histopathology evaluation

For histology evaluations, joints were fixed in formalin. They were measured with a caliper and then decalcified as described previously [2,3]. They were sectioned and stained with H & E. Sections were evaluated and scored according to the system mentioned by us before.

### Quantitation of Chlamydia in synovial tissues

We have adapted a clinical use ELISA kit, the IDEIA *PCE Chlamydia *from OXOID-Dako (Basingstoke,, UK) for the quantitation of *Chlamydia *in tissues [6]. Synovial tissues from the joints were carefully dissected out and homogenized. They were suspended in the transportation medium provided by the ELISA kit in a fashion as to normalize samples to the same wet weight per volume. All samples were frozen and stored at -70°C until assayed.

### Treatment of rats

NM was purchased from Sigma (St. Louis, MO, USA). The treatment scheme of Li *et al. *[7] for mice was followed. Daily intraperitoneal injections at a dose of 10 mg/kg body weight were made. NM was dissolved fresh in water each day just before use and then filtered sterilized before injection. The animals were briefly anesthetized with Isofurane (Zenoca Pharma, Missisauga, Ont., Canada), weighed and then the precise amount injected intraperitoneally. The injection schedule began on the day before *Chlamydia *infection, and then on a daily basis until the rats were sacrificed. Sterile water was injected into the untreated control rats.

### *In vitro *inhibition of *Chlamydia *proliferation

Lewis rat synovial fibroblast monolayers were set up on six well tissue culture plates. One hour prior to inoculation with *Chlamydia*, NM was added to the wells to give a range of concentrations from 0 to 200 μg/mL in duplicate wells. One hour after exposure to NM, the monolayers were infected at 10^5 ^CFU/well. The plates were spun down at 2 000 × G for 20 minutes to impact the bacteria onto the cells. Thereafter, plates were incubated at 37°C in a 5% CO_2 _incubator for 24 hours.

At the end of the incubation period, the monolayers were examined under phase contrast microscopy for *Chlamydia *inclusion bodies. Individual wells were harvested using cell scrapers and the contents frozen at -70°C until assayed.

The above IDEIA *PCE Chlamydia *kit from OXOID-Dako was also used for the quantitation of *Chlamydia *growth. Since the read out for this ELISA was in O.D., in order to combine different experimental runs, percent inhibition was calculated. The O.D. values from wells without NM was set at 0% inhibition.

The percentage difference from test wells with decreased O.D. values was calculated as percent inhibition.

## Results

### *In vitro *inhibition of *Chlamydia *proliferation

**Figure 1:**
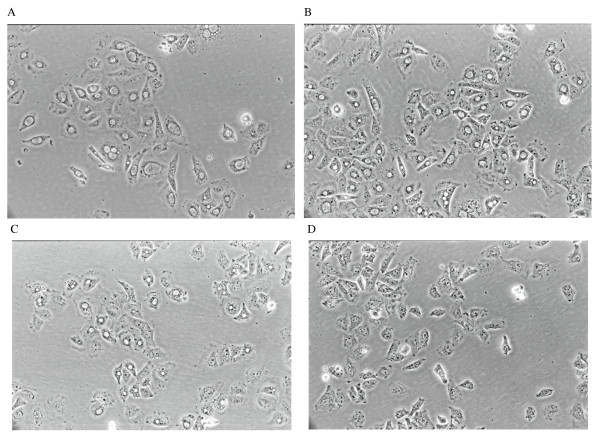
**Phase contrast photomicrographs of fibroblasts in culture, inoculated with chlamydia for 24 hours**. Panel **A**. No NM added, cells filled with large inclusion bodies. Panel **B**. 5 μg/mL of NM added, variations of inclusion sizes seen. Panel **C**. 10 μg/mL of NM added, inclusions are much smaller. Panel **D**. 50 μg/mL of NM added, no inclusion bodies present. NM, nafamostat mesylate.

Twenty four hour after fibroblast monolayers were inoculated with 10^5 ^CFU/well of *Chlamydia*, large inclusion bodies could be seen under phase contrast microscopy within the majority of the cells. When NM was introduced into the culture media, obvious effects could be observed at 5 μg/mL concentration. The sizes of the inclusion bodies became more variable and smaller ones could be seen (Figure 1). With increasing concentrations of NM the number of inclusions decreased and also became smaller. At 50 μg/mL and above, no inclusion bodies could be found.

**Figure 2:**
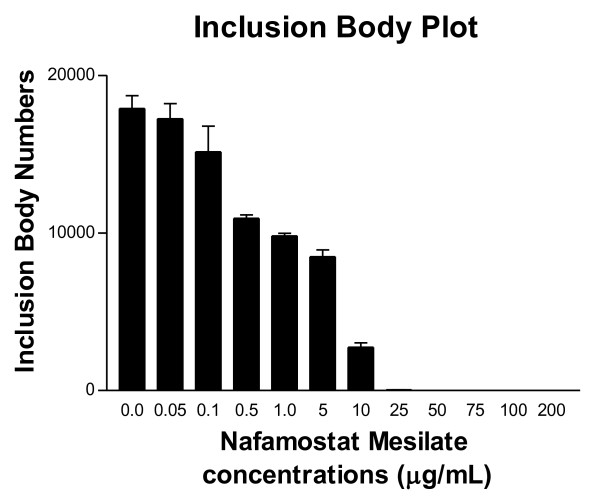
**The number of inclusion bodies decreased with increasing NM concentrations**. Under phase contrast microscopy, the number of inclusion bodies in 100 high power (40x) fields per well in duplicate wells for each NM concentration were numerated. Results represent data averaged from three separate experiments. NM, nafamostat mesylate.

Under phase contrast microscopy, the number of inclusion bodies in 100 high-power (40x) fields per well in duplicate wells for each NM concentration were carefully enumerated. Results are plotted in Figure 2 which represents data averaged from three separate experiments. Without NM in the media, the average number of inclusion bodies per well was 17,886 (± 1415). At 5 μg/mL, there were 8,490 (± 756) inclusions. At 25 μg/mL 35 inclusions were found and no inclusion bodies were observed at 50 μg/mL.

**Figure 3:**
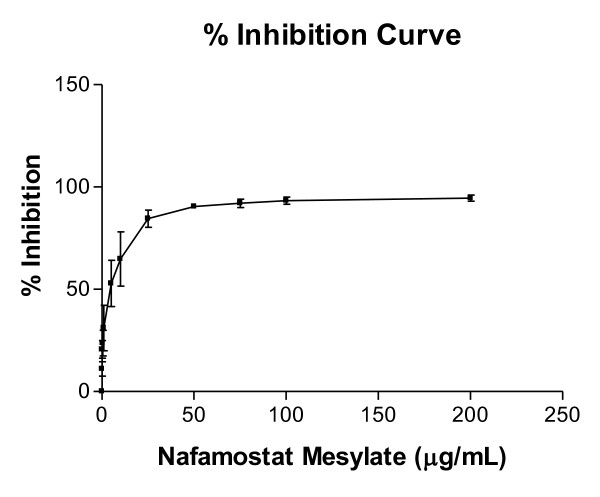
**Inhibition curve of chlamydia proliferation by ELISA**. The chlamydial antigens in each well along the concentration gradient were assayed by ELISA. Results are the average percent inhibitions of three experiments with duplicate wells for each concentration. ELISA, enzyme-linked immunosorbent assay; NM, nafamostat mesylate.

The chlamydial antigens in each well were assayed by ELISA along the concentration gradient (Figure 3). Results are expressed as the average percent inhibition of three separate experiments with duplicate wells for each concentration. It can be seen that NM is a very powerful inhibitor of *Chlamydia *proliferation. At 25 μg/mL inhibition was almost complete. This mirrored the inclusion body counts above.

### *Chlamydia *induced arthritis in rats

**Figure 4:**
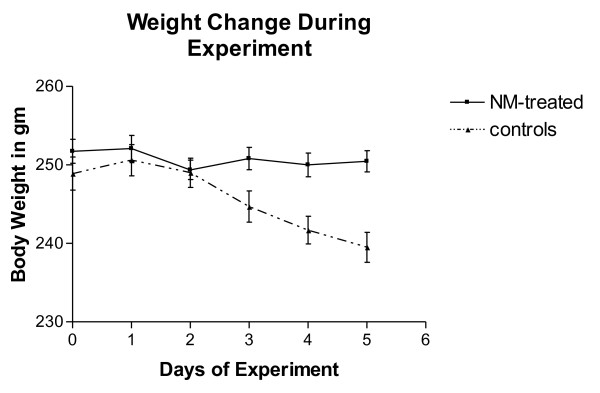
**Weight lost experienced by untreated control rats (*n *= 10) during the experimental period as compared to minimal change in the NM-treated rats (*n *= 10)**. N, number; NM, nafamostat mesylate.

As expected from our model of CtIA, the non-treated control rats began to experience symptoms of joint inflammation beginning on the second day after intra-articular infection with synoviocyte-packaged *Chlamydia*. By the end of the experiment four days later, all the injected joints were severely swollen. Animals experienced gradual weight lost for the period of the experiment beginning on Day 3 (Figure 4). On the other hand, the body weights of similarly infected rats receiving daily treatments with NM remained constant. The infected knee joints of these treated rats only experienced minimal swelling. At necropsy, *Chlamydia*-infected joints of the rats were measured. The average lateral width of the infected joints for the NM treated animals was 8.55 mm (sd ± 0.6578, *n *= 10) while that of the untreated control rats was significantly larger, 11.18 mm (sd ± 0.5672, *n *= 10) (*P *< 0.001).

### Histopathology

**Figure 5:**
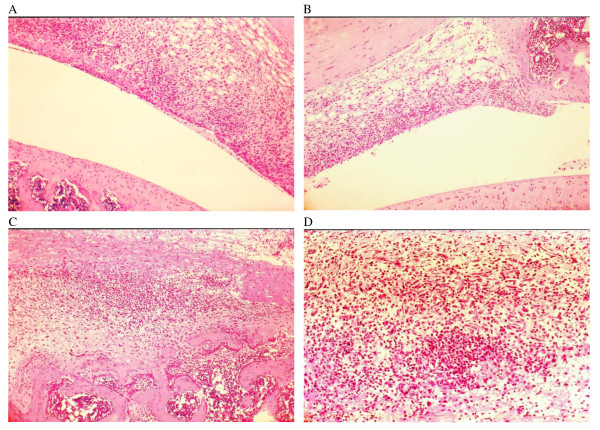
**H & E sections of Chlamydia-injected knee joints**. Panel **A **and **B **knee joints of NM-treated rats. Panel **C **and **D **knee joints of non-treated controls. In Panels A and B, there are moderate infiltrations of inflammatory cells into the synovium. However, the synovial space is clear and a considerable amount of the fatty tissues remains. The cartilage surfaces are mostly intact. On the other hand, in Panels C and D, inflammation is extensive, the whole architecture of the joints was destroyed. Cartilage tissues had been totally eroded away and the invading panus has entered the bone marrow space. Panel D also shows an area of early focus of necrosis. Original photomicrographs taken at 10× magnification. NM, nafamostat mesylate.

The knee joints of untreated control rats all had severe inflammatory changes with heavy infiltration of leukocytes, predominately neutrophils, consistent with our established experimental model (Figure 5). Soft synovial tissues were heavily hypertrophic with foci and necrosis, and there were extensive erosive changes. Most of the cartilage tissues showed structural damage with active panus formation. Panus could be seen invading through into the bone marrow spaces. Using the Yang-Hamilton grading scale, control knees had a mean score of 15.9 (± 1.45, *n *= 10). In contrast, infected knee joints from NM-treated rats showed much milder histopathology. The leukocyte infiltrations were moderate and the majority of the cells were mononuclear in nature. In areas where the infiltration is light, synovial fatty tissues could be seen. The synovial spaces were clearly defined and there were only minor erosive changes. The average Yang-Hamilton score for the test group is only 10.9 (± 2.45, *n *= 11). The difference between test and control scores is very significant at 9.79 × 10^-6^.

### Quantitation of *Chlamydia *in synovial tissues

**Figure 6:**
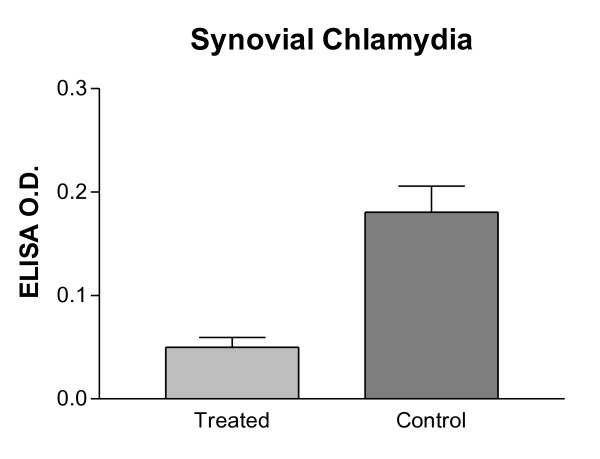
**Comparison of Chlamydia recovery from synovial tissues of NM-treated and control knees, as assayed by ELISA**. There is significantly more chlamydia in the synovial tissues of the non-treated control knees than in the NM treated ones (*P *= 0.003). ELISA, enzyme-linked immunosorbent assay; NM, nafamostat mesylate.

The amounts of chlamydial antigen within the synovial tissues of the infected knee joints were assayed by ELISA. As shown in Figure 6, it is obvious that there is significantly more *Chlamydia *in the synovial tissues of the non-treated control knees than in the NM treated joints. The mean O.D. values for the controls is 0.18 (± 0.05, *n *= 4) and for the NM-treated knees is 0.05 (± 0.02, *n *= 4). The difference is significant (*P *< 0.001).

## Discussion

Among the spondyloarthropathies, the role of infection as a triggering factor is best established in reactive arthritis (ReA) in which a sterile synovitis follows an extraarticular infection. ReA occupies the conceptual ground somewhere between septic arthritis and the classic autoimmune joint diseases such as RA [8]. Studies in ReA indicate that about 50% of such cases can be attributed to a specific pathogen by a combination of culture and serology, the predominant organisms being *Salmonella*, *Yersinia *and *Chlamydia*. It has increasingly been recognized that post-viral arthritis also constitutes an important subset of ReA. *C. trachomatis *has the strongest and most direct evidence for induction of ReA, with ReA occurring in 4% to 15% of those with chlamydial infections. Recent studies have demonstrated that current estimates for prevalence are likely underestimated as more than 60% of undifferentiated Spondyloarthritis represents post-*Chlamydia *ReA [9]. ReA is the paradigm of a rheumatic disease which reflects a dynamic interface between environmental triggers and genetic susceptibility. The fundamental distinctions between inflammatory joint disease as being autoimmune, autoinflammatory, or septic in nature are highlighted by the example of ReA [10].

NM is a serine protease inhibitor used clinically as an anti-inflammatory. As a protease inhibitor, it is effective in neutralizing the enzymatic activities of activated complement components such as C1r, C1s, C3 and C5 convertases [4,7,11-15]. It is also effective in inhibiting elements in the alternate pathway such as Factors B and D [11,13,14]. Similarly it is able to counter the activation of key molecules in the coagulation cascade such as thrombin and plasmin [14,16,17].

As an anti-inflammatory agent NM appears to prevent granulocyte and phagocytic cell accumulations into damaged tissues [11,12,14]. Immunologically, NM is also known to suppress the production of cytokines IFNγ, TNF, IL17, IL4, IL5, IL6 and IL13 [7,16,17]. NM has also been suggested as an anti-tumor agent since it interferes with NF-κB activation in cancer cell lines [18] and in experimental rodent models [19].

Because of its broad range of actions as a protease inhibitor, NM has been used experimentally in diverse settings. Ishizak *et al. *[12] used NM on a model of murine allergy while Hagiwara *et al. *[17] used it to prevent lung injury induced by LPS. Bonte *et al. *[11] and Schwertz *et al. *[14] used NM to reduce cardiac injury following experimental ischemia. Miyagi *et al. *[16] reported a good outcome in experimental liver transplant. Promising results were also reported in a rodent experimental autoimmune encephalomyelitis model [7] as well as in a rabbit model of autoimmune Guillain-Barre syndrome [13]. NM has also been used to treat adjuvant arthritis in rats to reduce the severity of the arthritis [15]. Other than this early report in adjuvant arthritis, the effect of NM in other types of arthritis has not been evaluated.

Our data provide the first evidence that NM has important antimicrobial properties. This could have important implications for treatment options for clinical *Chlamydia*-induced arthritis. At present the treatment options are few [1]. There has recently been completed a randomized, placebo-controlled study of combination antibiotics for chronic *Chlamydia*-induced arthritis [20]. This has created renewed interest in examining agents that might have both anti-inflammatory and antimicrobial effects. Future studies will address time kinetics of different schedules of NM in the experimental model. Our findings in the experimental model indicate that NM would be an appealing candidate for such a therapeutic indication, and would be worthy of further investigation in this regard.

## Conclusions

NM is a protease inhibitor not previously recognized to possess antimicrobial properties. The present study demonstrates for the first time that NM exerts a significant impact on *C. trachomatis*-induced arthritis. These findings suggest that such an approach may prove clinically useful in chronic reactive arthritis.

## Abbreviations

CtIA: *Chlamydia trachomatis*-induced arthritis; CFU: colony-forming unit; ELISA: enzyme-linked immunosorbent assay; H & E: haematoxylin and eosin; IB: inclusion bodies; IFN: interferon; IL: interleukin; LEW: Lewis rats; NM: Nnfamostat mesylate; O.D.: optical density; RA: rheumatoid arthritis; ReA: reactive arthritis; TNF: tumor necrosis factor.

## Competing interests

The authors declare that they have no competing interests.

## Authors' contributions

BC carried out the *in vitro *and *in vivo *experiments. RI and BC jointly developed the first draft and the final revisions to the manuscript. Both authors have read and approved the final manuscript.
